# Supplementary figures and images for: Metabolic pathway alterations in microvascular endothelial cells in response to hypoxia
Source: PLoS One. 2020 Jul 9;15(7):e0232072. doi: 10.1371/journal.pone.0232072 (PMC7347218; doi:10.1371/journal.pone.0232072)

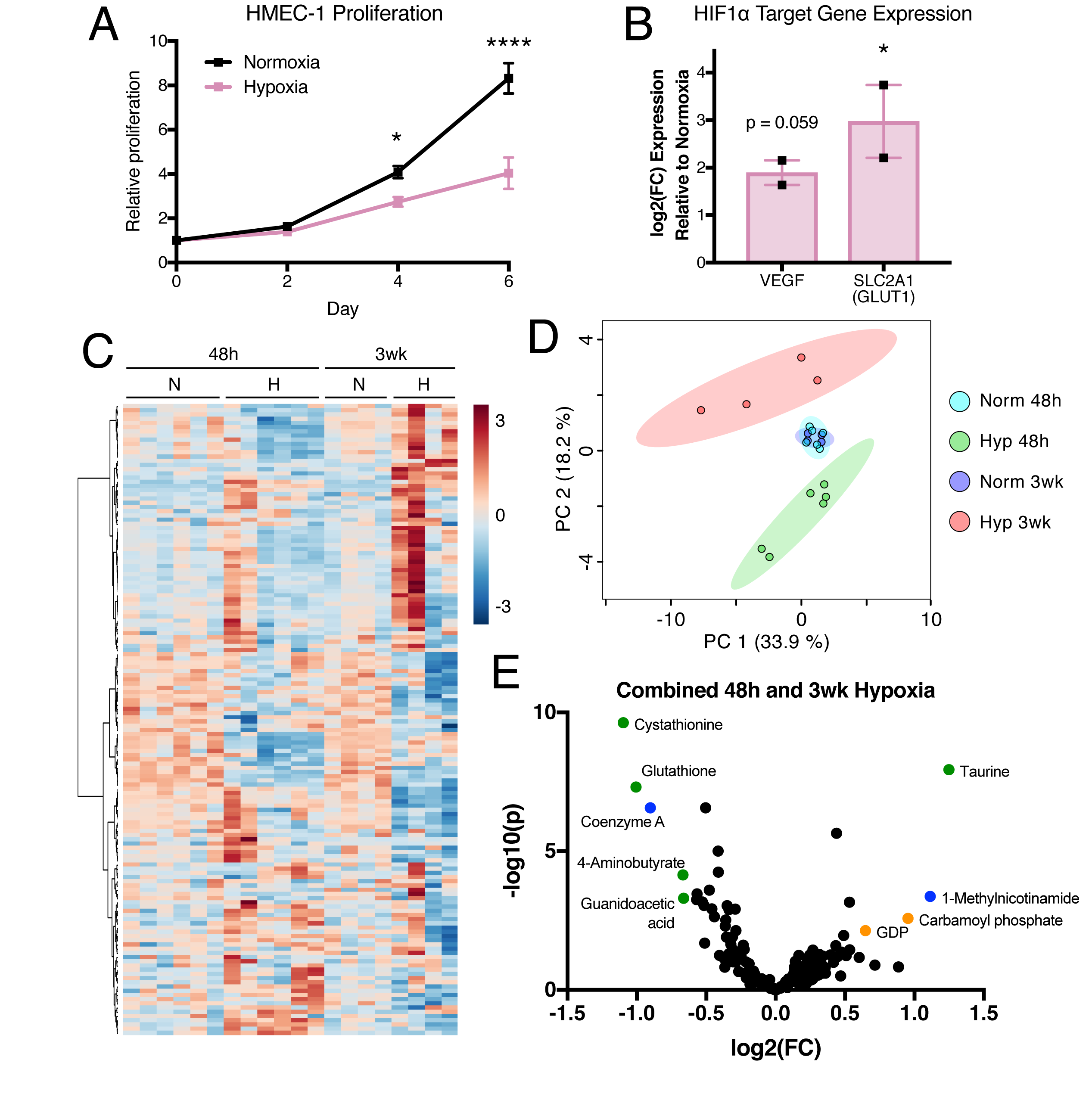

Supplement: S1 Fig — (A) HMEC-1 cells were grown in normoxia or hypoxia for six days. Proliferation of the cells was determined using sulforhodamine B staining at the indicated time points, N = 4 biological replicates. (B) mRNA abundance of HIF1α target genes from 3-week metabolomics-matched RNA samples were measured by qRT-PCR and is expressed as log(2) fold change relative to HMEC-1 cells cultured in normoxia, N = 2 biological replicates. (C) Fold change metabolite abundance over normoxia control for 150 metabolites detected in all samples as measured by LC-MS/MS of HMEC-1 cells grown in hypoxia for 48 hrs and 3 weeks. (D) Principal component analysis of data from C. (E) Effect size and significance by t-test of metabolite changes measured in C. Colored circles indicate p < 0.05 and fold change > 1.5. Colors indicate pathways: green, amino acid metabolism; orange, nucleotide metabolism; blue, other. All error bars represent SEM. Significance by two-way ANOVA except E as described above. *, p < 0.05; ****, p < 0.0001. (TIF) [file pone.0232072.s001.tif]

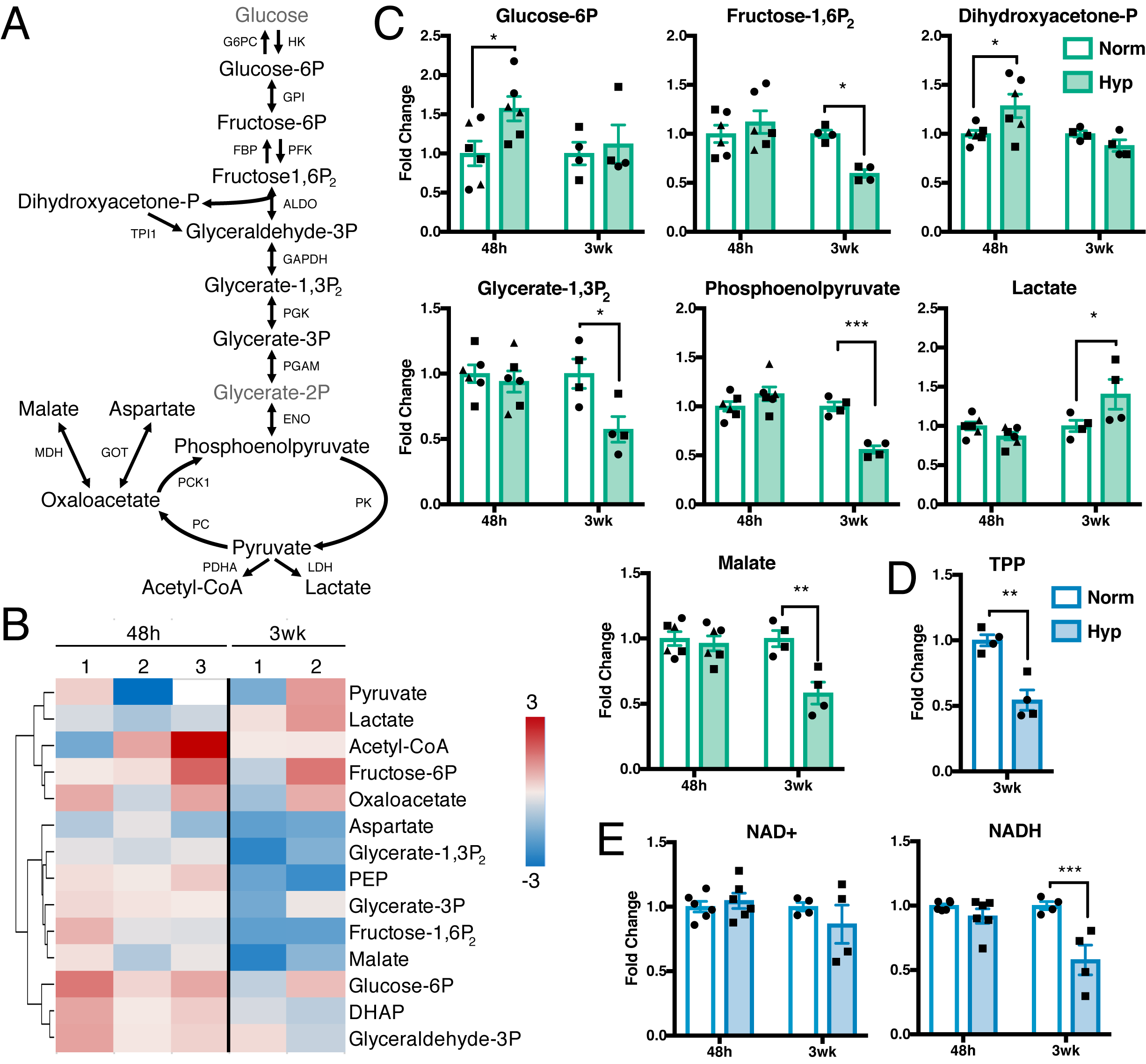

Supplement: S2 Fig — (A) Metabolites and enzymes in glycolysis. Metabolites in gray not measured or detected. (B) Fold change metabolite abundance over normoxia control for metabolites in pathway A detected in more than one sample. White space indicates metabolite not detected. (C) Significantly altered metabolites from B. (D-E) Fold change abundance compared to normoxia of cofactors for glycolytic enzymes. All error bars represent SEM, different symbols represent biological replicates. Significance by two-way ANOVA except D by unpaired t-test. *, p < 0.05; **, p < 0.01; ***, p < 0.001. Enzyme abbreviations: ALDO, aldolase; ENO, enolase; FBP, fructose-1, 6-bisphosphatase; G6PC, glucose-6-phosphatase; GAPDH, glyceraldehyde 3-phosphate dehydrogenase; GOT, aspartate aminotransferase; GPI, glucose-6-phosphate isomerase; HK, hexokinase; LDH, lactate dehydrogenase; MDH, malate dehydrogenase; PC, pyruvate carboxylase; PCK1, phosphoenolpyruvate carboxykinase; PFK, phosphofructose kinase; PGAM, phosphoglycerate mutase; PGK, phosphoglycerate kinase; PK, pyruvate kinase. Metabolite abbreviations: CoA, coenzyme A; DHAP, dihydroxyacetone phosphate; NAD+, oxidized nicotinamide adenine dinucleotide; NADH, nicotinamide adenine dinucleotide; P, phosphate; P2, bisphosphate; PEP, phosphoenolpyruvate; TPP, thiamine pyrophosphate. (TIF) [file pone.0232072.s002.tif]

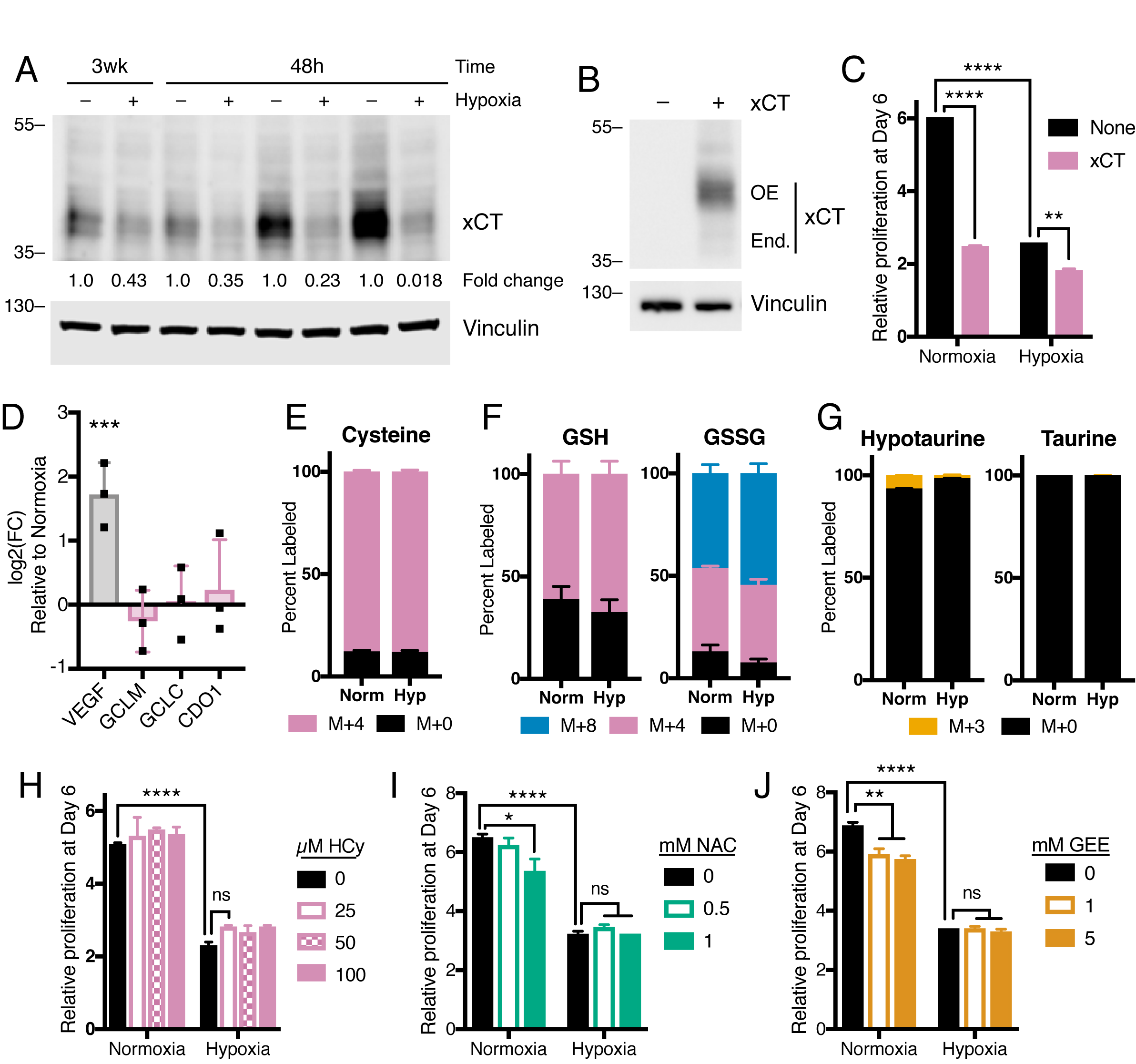

Supplement: S3 Fig — (A) HMEC-1 cell lysates were prepared from all 48-hr and one 3-week metabolomics-matched protein samples and immunoblotted for xCT expression. Relative xCT expression in hypoxia was calculated using Bio-Rad Image Lab and is expressed as fold change relative to paired normoxia sample. (B) Lysates were prepared from naïve HMEC-1 cells or HMEC-1 cells overexpressing xCT and immunoblotted for xCT expression. (C) Naïve HMEC-1 cells or HMEC-1 cells overexpressing xCT were grown in normoxia or hypoxia for six days, and the proliferation of the cells was determined using SRB staining, N = 3 technical replicates. (D) mRNA abundance of cysteine metabolizing enzymes in HMEC-1 cells after 48 hrs of normoxia or hypoxia exposure was measured by qRT-PCR and is expressed as log(2) fold change relative to HMEC-1 cells cultured in normoxia, N = 3 biological replicates. (E-G) Mass isotopomer analysis by LC-MS/MS of (E) cysteine, (F) reduced and oxidized glutathione, and (G) hypotaurine and taurine in HMEC-1 cells cultured in normoxia or hypoxia for 48 hrs, the last 16 hrs of which in medium containing 165 μM U-13C315N-cysteine, N = 2 technical replicates. (H-J) HMEC-1 cells were grown in normoxia or hypoxia for six days in the presence or absence of (H) homocystine (Hcy), (I) N-acetyl cysteine (NAC), or (J) glutathione ethyl ester (GEE). Proliferation of the cells was determined using SRB staining, N = 3 technical replicates. All error bars represent SEM. Significance by two-way ANOVA. *, p < 0.05; **, p < 0.01; ***, p < 0.001; ****, p < 0.0001. (TIF) [file pone.0232072.s003.tif]

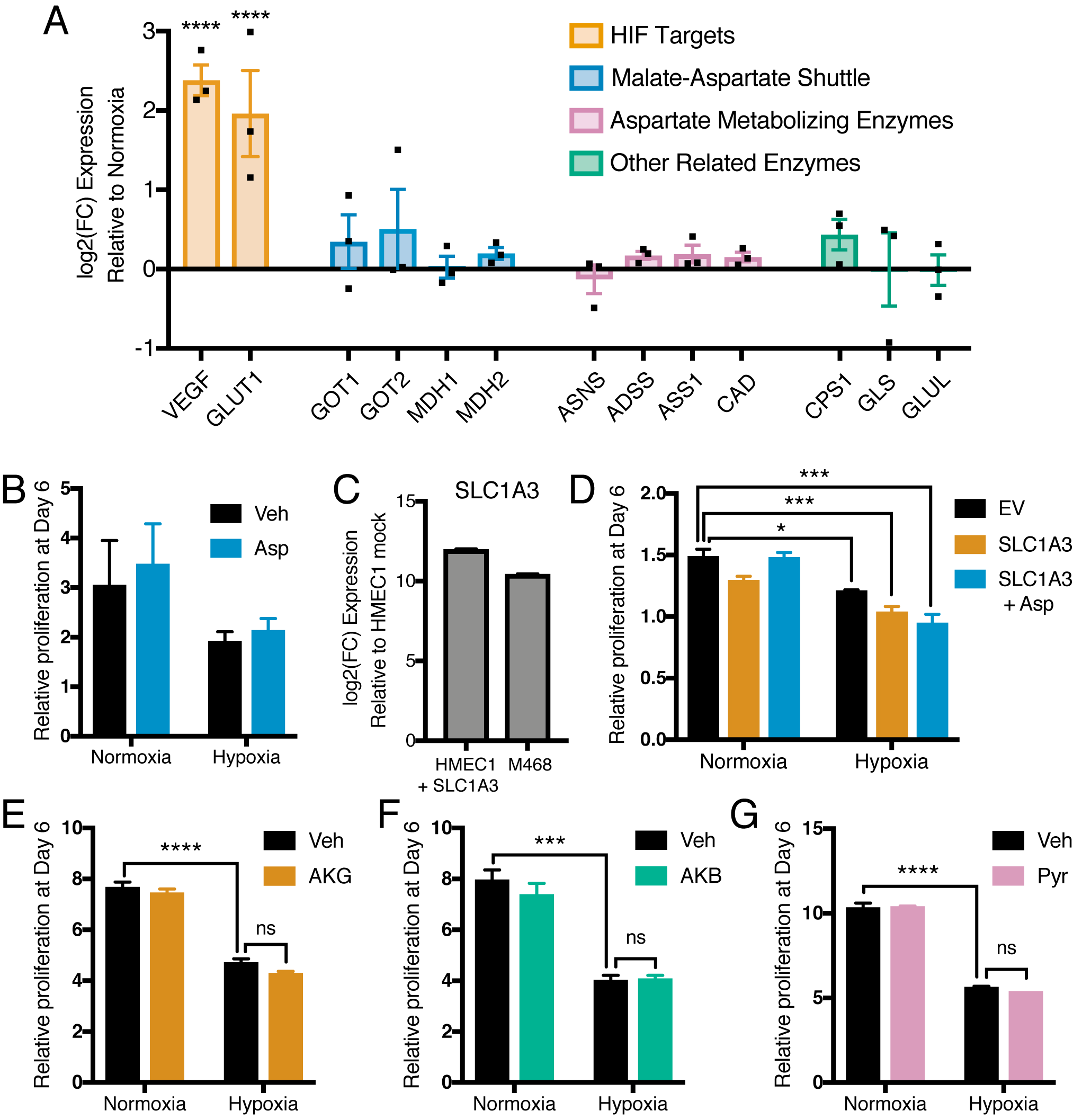

Supplement: S4 Fig — (A) mRNA abundance of aspartate metabolism-associated enzymes in HMEC-1 cells after 48 hrs of normoxia or hypoxia exposure was measured by qRT-PCR and is expressed as log(2) fold change relative to HMEC-1 cells cultured in normoxia, N = 3 biological replicates. (B) HMEC-1 cells were grown in normoxia or hypoxia for six days in the presence or absence of 20 mM aspartate. Proliferation of the cells was determined using SRB staining, N = 2 biological replicates. (C) mRNA abundance of SLC1A3 in HMEC-1 cells overexpressing SLC1A3 or naïve MDA-MB-468 breast cancer cells was measured by qRT-PCR and is expressed as log(2) fold change relative to naïve HMEC-1 cells, N = 3 technical replicates. (D) HMEC-1 cells expressing an empty vector or SLC1A3 were grown in normoxia or hypoxia for six days in the presence or absence of 150 μM aspartate in normoxia or hypoxia. Proliferation of the cells was determined using SRB staining, N = 3 technical replicates. HMEC-1 cells were grown in normoxia or hypoxia for six days in the presence or absence of (E) 1 mM dimethyl α-ketoglutarate (AKG), (F) 1 mM α-ketobutyrate (AKB), (G) 2 mM pyruvate. Proliferation of the cells was determined using SRB staining, N = 3 technical replicates. All error bars represent SEM. Significance by two-way ANOVA. *, p < 0.05; ***, p < 0.001; ****, p < 0.0001. (TIF) [file pone.0232072.s004.tif]

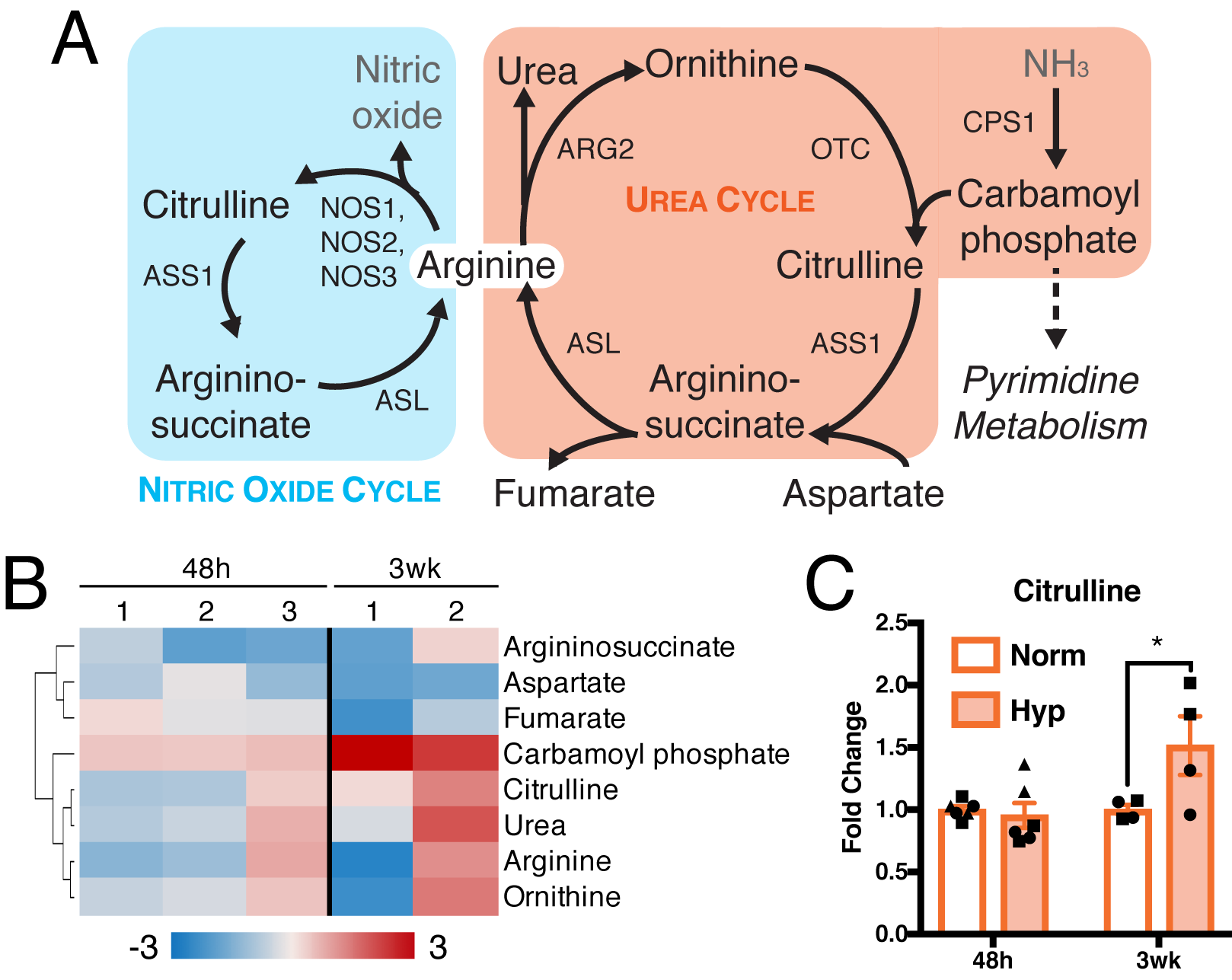

Supplement: S5 Fig — (A) Metabolites and enzymes in arginine metabolism. Metabolites in gray not measured or detected. (B) Fold change metabolite abundance over normoxia control for metabolites in pathway A detected in more than one sample. (C) Significantly altered metabolite from B not shown in prior figures. Significance by two-way ANOVA. All error bars represent SEM, different symbols represent biological replicates. *, p < 0.05. Abbreviations: ARG2, arginase 2; ASL, argininosuccinate lyase; ASS1: argininosuccinate synthase; CPS1, carbamoyl phosphate synthetase; NOS1/2/3, nitric oxide synthase 1/2/3; OTC, ornithine transcarbamylase. (TIF) [file pone.0232072.s005.tif]
