# Supplementary material for: Metabolic pathway alterations in microvascular endothelial cells in response to hypoxia
Source: PLoS One. 2020 Jul 9;15(7):e0232072. doi: 10.1371/journal.pone.0232072 (PMC7347218; doi:10.1371/journal.pone.0232072)

Fig 1A
HIF1a panel

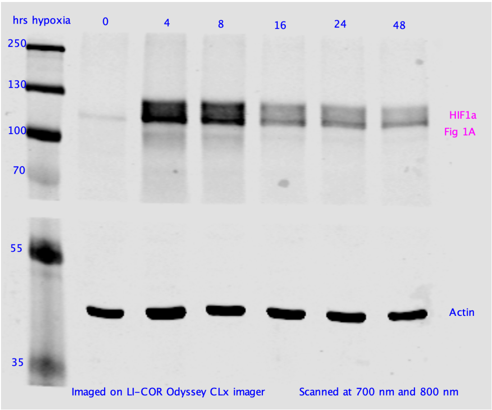

Fig 1A
Actin panel

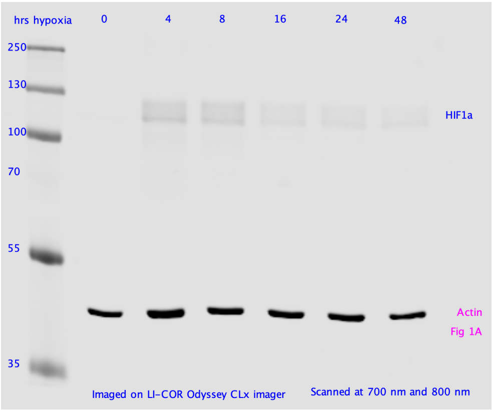

S3 Fig A
unmerged xCT panel

version in final figure

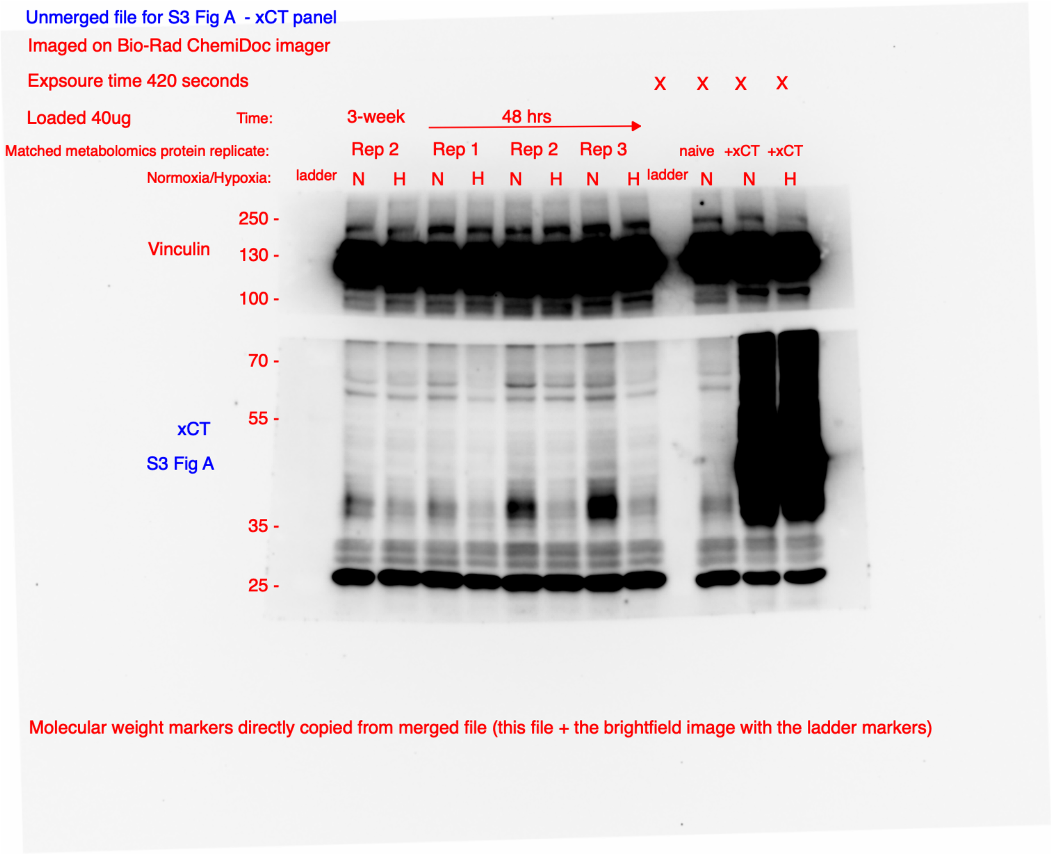

S3 Fig A
merged xCT panel

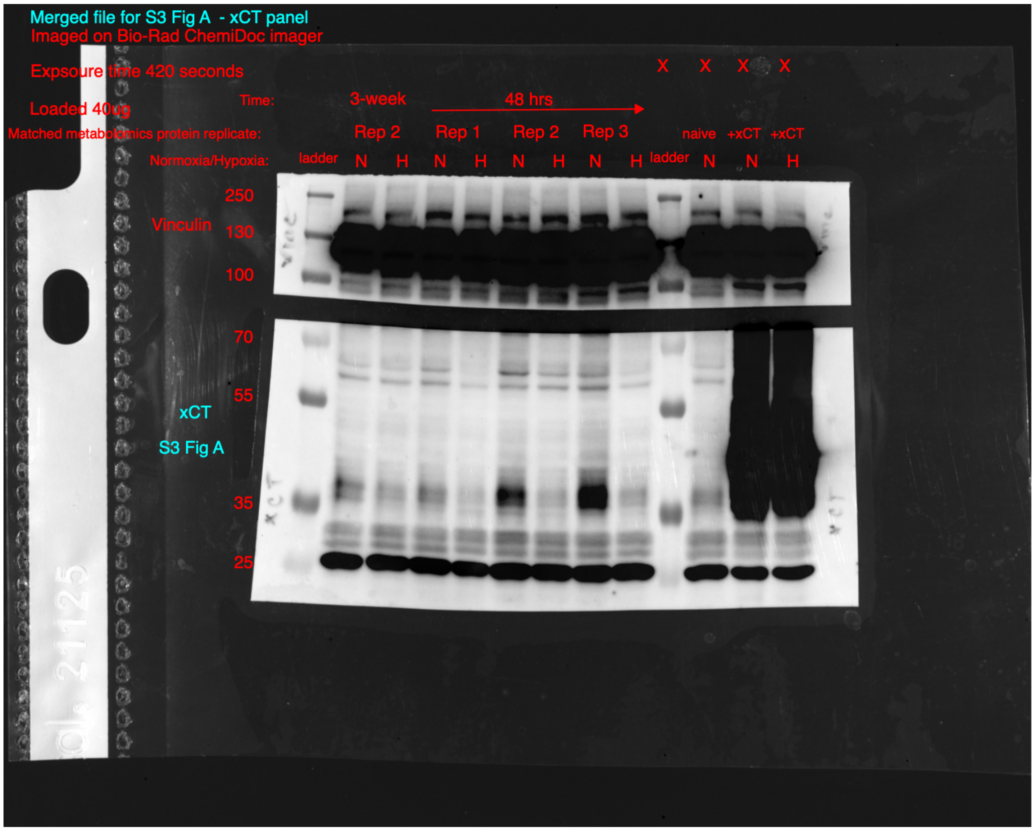

S3 Fig A
unmerged vinculin panel

version in final figure

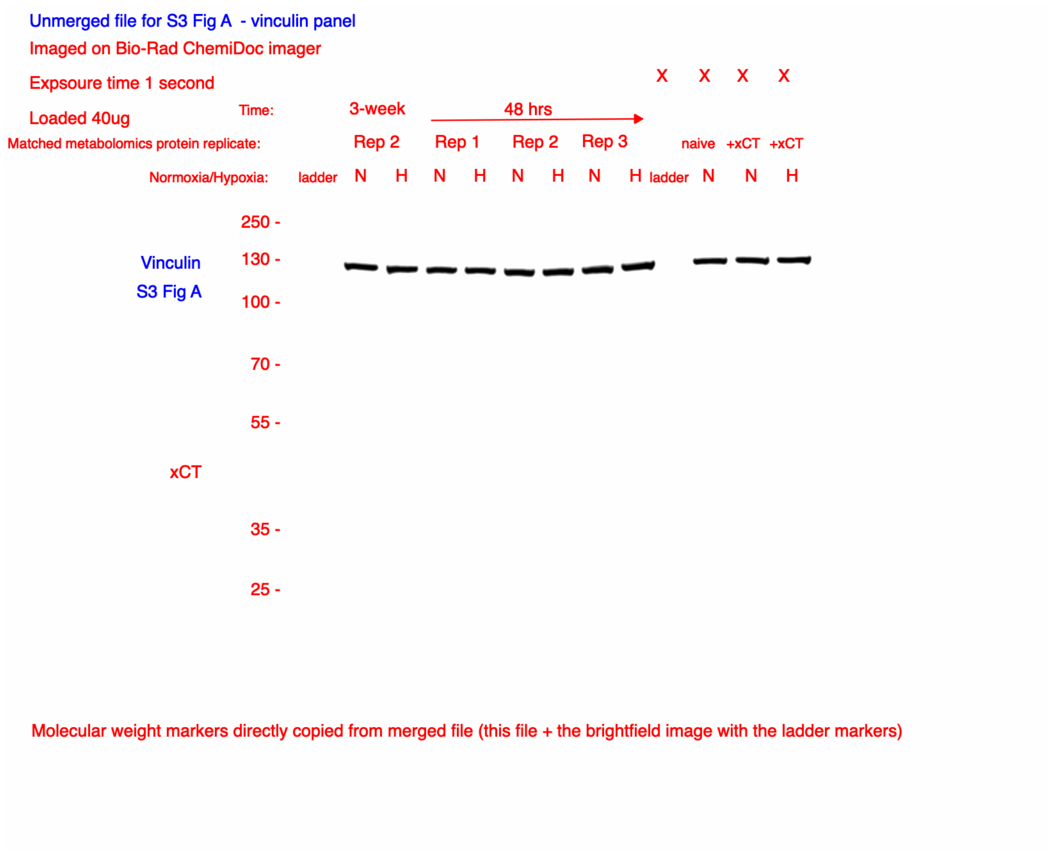

S3 Fig A
merged vinculin panel

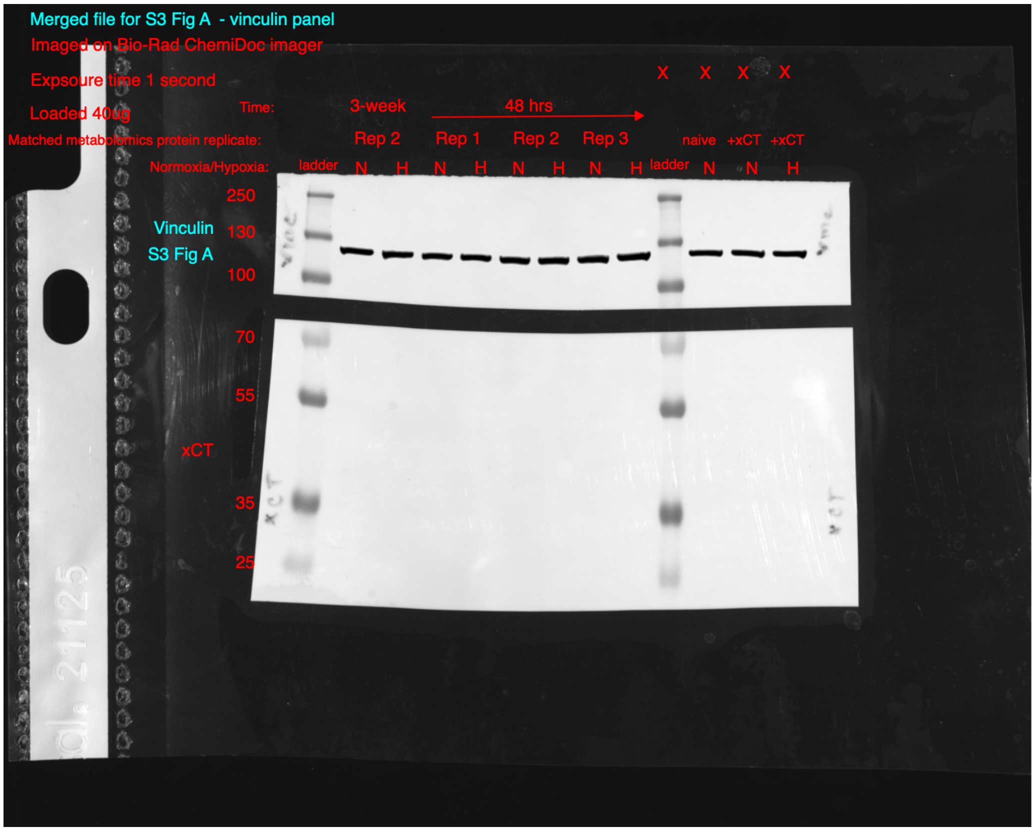

S3 Fig B
unmerged xCT panel

version in final figure

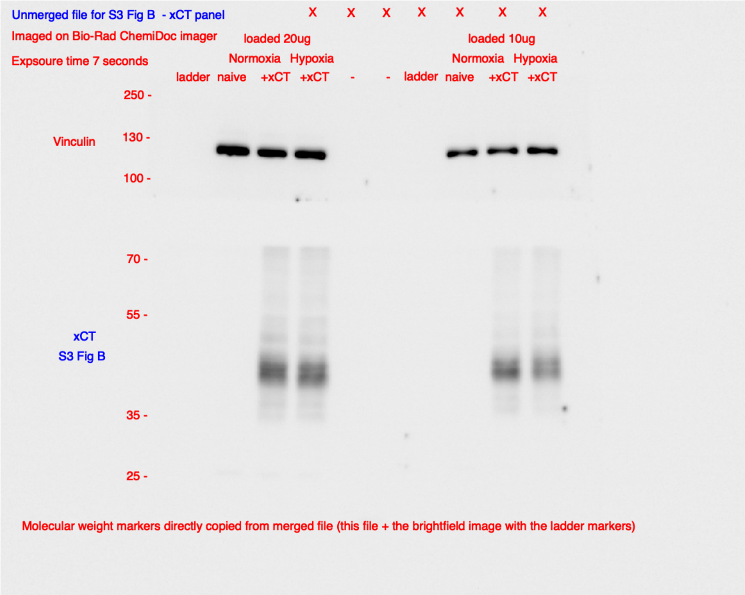

S3 Fig B
merged xCT panel

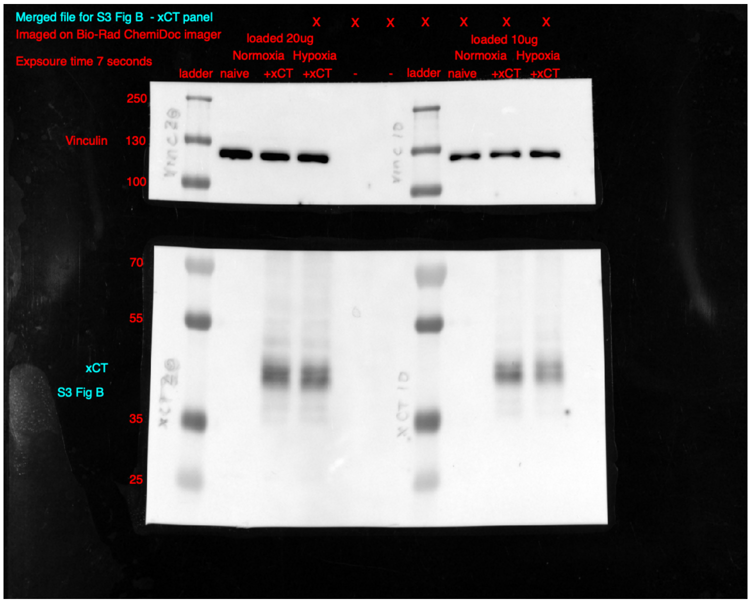

S3 Fig B
unmerged vinculin panel
version in final figure

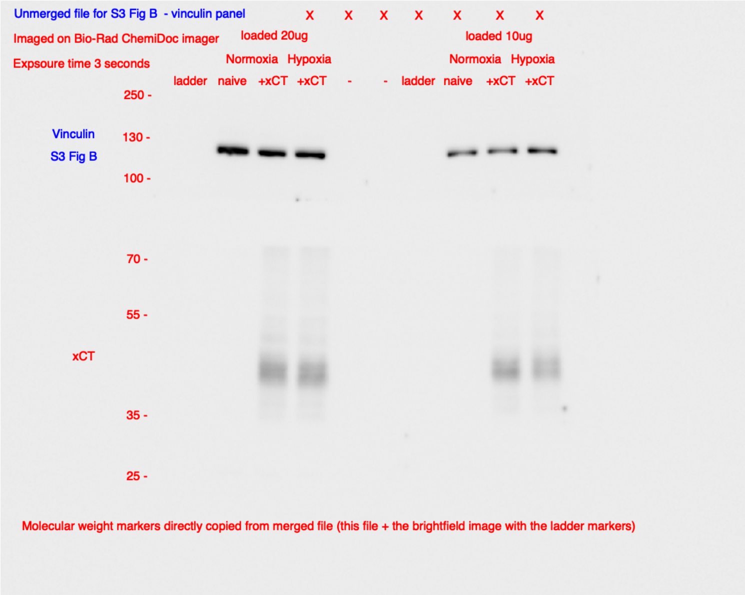

S3 Fig B
merged vinculin panel

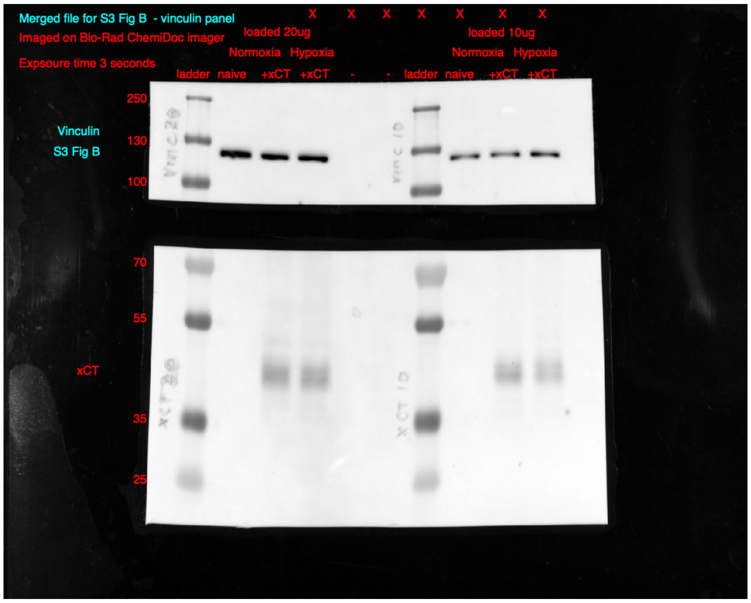

Supplement: S1 Raw images — (PDF) [file pone.0232072.s006.pdf]
