# Supplementary material for: Metabolic pathway alterations in microvascular endothelial cells in response to hypoxia
Source: PLoS One. 2020 Jul 9;15(7):e0232072. doi: 10.1371/journal.pone.0232072 (PMC7347218; doi:10.1371/journal.pone.0232072)
Supplement: Attachment — Submitted filename: Response to Reviewers_FINAL.pdf [file pone.0232072.s010.pdf]

**PONE-D-20-09862**
**Metabolic pathway alterations in microvascular endothelial cells in response to hypoxia**
**PLOS ONE**

Reviewer #1: We thank you for your positive review of our study.

Reviewer #2: We thank you for suggesting an additional discussion of aerobic versus anaerobic metabolism, and your recommendation of a relevant article for citation. This is an excellent point. We have now included such a discussion on pages 20 and 22, and added the relevant citation. Furthermore, we have clarified that all underlying data is in fact available as Supporting Information, including full blot images as S1_raw_images.
